# RETRACTION: Effects of Evidence‐Based Nursing on Surgical Site Wound Infections in Patients Undergoing Surgery for Liver Cancer: A Meta‐Analysis

**DOI:** 10.1111/iwj.70279

**Published:** 2025-03-03

## Abstract

**RETRACTION:** ShiY.‐H. , WangQ.‐J. , HuangL.‐Z. , LiN. , RenX.‐T. , and BiS.‐M. , “Effects of Evidence‐Based Nursing on Surgical Site Wound Infections in Patients Undergoing Surgery for Liver Cancer: A Meta‐Analysis,” International Wound Journal 21, no. 1 (2024): e14545, 10.1111/iwj.14545.38272814 PMC10791573

The above article, published online on 16 January 2024, in Wiley Online Library (http://onlinelibrary.wiley.com/), has been retracted by agreement between the journal Editor in Chief, Professor Keith Harding; and John Wiley & Sons Ltd. Following an investigation by the publisher, all parties have concluded that this article was accepted solely on the basis of a compromised peer review process. The editors have therefore decided to retract the article. The authors did not respond to our notice regarding the retraction.

**RETRACTION:** Y.‐H. Shi , Q.‐J. Wang , L.‐Z. Huang , N. Li , X.‐T. Ren , and S.‐M. Bi , “Effects of Evidence‐Based Nursing on Surgical Site Wound Infections in Patients Undergoing Surgery for Liver Cancer: A Meta‐Analysis,” International Wound Journal 21, no. 1 (2024): e14545, 10.1111/iwj.14545.38272814 PMC10791573

The above article, published online on 16 January 2024, in Wiley Online Library (http://onlinelibrary.wiley.com/), has been retracted by agreement between the journal Editor in Chief, Professor Keith Harding; and John Wiley & Sons Ltd. Following an investigation by the publisher, all parties have concluded that this article was accepted solely on the basis of a compromised peer review process. The editors have therefore decided to retract the article. The authors did not respond to our notice regarding the retraction.

